# Editorial: Insights in applied neuroimaging: 2021

**DOI:** 10.3389/fneur.2022.1048674

**Published:** 2022-11-02

**Authors:** Jan Kassubek

**Affiliations:** Department of Neurology, University of Ulm, Ulm, Germany

**Keywords:** neuroimaging (anatomic and functional), brain disease and dynamics, multimodality, brain age, technical development

The annual “Insights in” Research Topic series in *Frontiers in Neurology* aims to collect studies that represent current challenges and discoveries, recent advances, and future perspectives in the respective field, i.e., in Applied Neuroimaging for this Research Topic. Contributions are considered to mirror the state of the art and to address major accomplishments that have been achieved. In the field of neuroimaging applications, both technical novelties, especially with respect to both data acquisition protocols including multimodal combinations and data analysis with innovation in novel (unbiased) algorithms and data integration, exploring the contribution of neuroimaging to complex clinical challenges. This Research Topic aims to specifically support the aim of the section to report clinical and neuroscientific research with all imaging modalities, providing a forum for the promising and rapidly advancing field of neuroimaging applications to the advanced structural and functional mapping of the nervous system (1).

In the Research Topic „Insights in Applied Neuroimaging 2021”, the multifaceted advances of current neuroimaging applications are represented in the publications according to this outline. Kenda et al. address the challenge of the prognostication of neurological outcome after cardiac arrest by CT and to this end, assessed the gray white matter ratio in the head CTs of 95 cardiac arrest patients both by human raters and by a recently published computer algorithm. They observed a very good interrater agreement between human raters with different levels of expertise and the computer algorithm, however, with considerable interrater variability in individual patients. The authors conclude that the results emphasize the need for strategies to standardize quantitative head CT analysis and for multimodal prognostication in general. In a study in 247 healthy subjects targeting to set up a reference data set, Sun Z., et al. used automatic vessel segmentation, centerline tracking, and phase mapping on MR angiography to investigate age and gender effects on brain-supplying neck arteries regarding tortuosity and flow changes. By this approach, quantifiable age-related morphological and hemodynamic alterations in the investigated arteries could be demonstrated, including the differences between female and male patients. Also in healthy subjects, Boban et al. studied physiological brain aging by a correlation analysis of four white matter diffusivity measures in diffusion tensor imaging (DTI) data with chronological age and education levels. All DTI metrics showed significant correlations with the advancing age of the participants, however, this involved largely different regional patterns and the authors concluded that different patterns of degradation during aging are true for different brain fiber tracts and that not one single of the currently available theories can globally explain age-related changes in the brain. In another study taking advantage of a fully automated MRI analysis technology, i.e., AccuBrain^TM^ for segmentation and quantitative volumetry, Sun W., et al. investigated 242 patients with cerebral small vessel disease and observed that the disease was associated with widespread cerebral atrophy including the lobes and that the volumes of periventricular white matter hyperintensities and medial temporal atrophy were independent predictors of cognitive decline. Ioachim et al. investigated the clinical challenge of chronic pain associated with fibromyalgia by task-based functional MRI with a pain-related paradigm; they observed differences in pain processing between people with fibromyalgia and healthy controls and that this altered pain processing may be linked to changes in both descending pain regulation and autonomic regulation, even while the participants are only anticipating the pain, thus advancing the understanding of fibromyalgia. By use of resting state functional MRI (rsfMRI) within a multiparametric protocol at ultra-high field of 7T, Morrison et al. analyzed the dorsal attention, salience, and frontoparietal networks in young patients after cranial radiation therapy for a brain tumor. Compared to controls, patients exhibited widespread hyperconnectivity, similar modularity, and significantly increased efficiency and network variability, correlated with memory performance, suggesting that these rsfMRI metrics might be promising imaging-based markers for monitoring the cognitive side effects of radiation therapy. An MRI-based study on brain changes associated with COVID-19 neurological manifestations was contributed by Napolitano et al., who investigated cerebral microbleeds by susceptibility-weighted imaging (including a semi-automatic processing procedure) in 63 patients during the first wave in Italy. Cerebral microbleeds were a frequent finding in hospitalized patients with COVID-19 and neurological manifestations, with a specific pattern of distribution (i.e., prominent callosal and juxtacortical involvement) and seemed to be related to pro-inflammatory status. In a technical report by a multi-national study group, Bouget et al. investigated standardized and automatic methods (i.e., Raidionics and Raidionics-Slicer within the AGU-Net architecture) for tumor detection and assessment of tumor characteristics in MRI in the most occurring brain tumor types (glioblastomas, lower grade gliomas, meningiomas, metastases) in up to 4,000 patients‘ data. The detailed performance assessment enabled the identification of the most relevant metrics from a large panel, and for clinical practice, tumor segmentation could be performed in less than a minute. The generation of a standardized clinical report took less than 15 min — *nota bene*, all trained models have been made publicly available (open-access) together with the source code for the software solutions and validation metrics computation. In the perspective article by Juengling et al., an international group of authors reviewed the current state of the art in the application of simultaneous PET/MRI to motor neuron disease (like amyotrophic lateral sclerosis) and how this combination of advanced neuroimaging modalities can guide in characterizing this neurodegenerative disease *in vivo* by the complementary information on disease pathology. Yeo et al. address the relationship between white matter hyperintensities in MRI and right-to-left shunt in migraine patients by a systematic review and meta-analysis (of 8 observational studies comprising 1,125 patients), and they reported that in migraine, the right-to-left shunt was significantly associated with the presence of white matter hyperintensities. Finally, in a case report (comparative case study) by Golden et al., two patients with fibrous dysplasia and similar craniofacial lesion burden were investigated by ^18^F-sodium fluoride PET/CT and multiparametric MRI, and the detailed phenotypic characterization incorporating the advanced imaging approach guided the understanding of the variable experiences with pain in craniofacial fibrous dysplasia.

These contributions demonstrate both technical and clinical “insights” on the application of advanced neuroimaging, comprising, on the one hand, various technical approaches with computed tomography, and multiparametric MRI including structural imaging (atrophy assessment, white matter alterations/lesions, tumor lesion, and susceptibility imaging), microstructural diffusion-based imaging, and functional imaging (both task-based and resting-state intrinsic connectivity-based), and multimodality approaches (PET/MRI and PET/CT). On the other hand, studies in healthy (*N* = 2) and diseased brains (*N* = 9) are included, the latter covering a very broad pathological spectrum from vascular diseases and neurodegeneration, COVID-19, and pain disorders to multi-organ orphan diseases. Finally, single-center cohort studies and multi-site collaborations of researchers are represented, together with meta-analysis data. Although heterogeneous in their approaches, the contributions to this Research Topic all target the integration of research on clinical and neuroscientific grounds with all modalities of neuroimaging and advanced postprocessing.

## Author contributions

The author confirms being the sole contributor of this work and has approved it for publication.

## Conflict of interest

The author declares that the research was conducted in the absence of any commercial or financial relationships that could be construed as a potential conflict of interest.

## Publisher's note

All claims expressed in this article are solely those of the authors and do not necessarily represent those of their affiliated organizations, or those of the publisher, the editors and the reviewers. Any product that may be evaluated in this article, or claim that may be made by its manufacturer, is not guaranteed or endorsed by the publisher.
